# Caretaker knowledge, attitudes, and practices (KAP) and carriage of extended-spectrum beta-lactamase-producing *E. coli* (ESBL-EC) in children in Quito, Ecuador

**DOI:** 10.1186/s13756-020-00867-7

**Published:** 2021-01-06

**Authors:** Rachel Marusinec, Kathleen M. Kurowski, Heather K. Amato, Carlos Saraiva-Garcia, Fernanda Loayza, Liseth Salinas, Gabriel Trueba, Jay P. Graham

**Affiliations:** 1 Berkeley School of Public Health, University of California, Berkeley, CA USA; 2 Microbiology Institute, Colegio de Ciencias Biologicas Y Ambientales, Universidad San Francisco de Quito, Quito, Ecuador

**Keywords:** Antibiotic resistance, Ecuador, Children, *Escherichia coli*, Extended-spectrum beta-lactamase, ESBL, ESBL-EC, Knowledge, attitudes and practices, KAP

## Abstract

**Background:**

The rapid spread of extended-spectrum beta-lactamase-producing *E. coli* (ESBL-EC) is an urgent global health threat. We examined child caretaker knowledge, attitudes, and practices (KAP) towards proper antimicrobial agent use and whether certain KAP were associated with ESBL-EC colonization of their children.

**Methods:**

Child caretakers living in semi-rural neighborhoods in peri-urban Quito, Ecuador were visited and surveyed about their KAP towards antibiotics. Fecal samples from one child (less than 5 years of age) per household were collected at two time points between July 2018 and May 2019 and screened for ESBL-EC. A repeated measures analysis with logistic regression was used to assess the relationship between KAP levels and child colonization with ESBL-EC.

**Results:**

We analyzed 740 stool samples from 444 children living in households representing a range of environmental conditions. Of 374 children who provided fecal samples at the first household visit, 44 children were colonized with ESBL-EC (11.8%) and 161 were colonized with multidrug-resistant *E. coli* (43%). The prevalences of ESBL-EC and multidrug-resistant *E. coli* were similar at the second visit (11.2% and 41.3%, respectively; N = 366). Only 8% of caretakers knew that antibiotics killed bacteria but not viruses, and over a third reported that they “always” give their children antibiotics when the child’s throat hurts (35%). Few associations were observed between KAP variables and ESBL-EC carriage among children. The odds of ESBL-EC carriage were 2.17 times greater (95% CI: 1.18–3.99) among children whose caregivers incorrectly stated that antibiotics do not kill bacteria compared to children whose caregivers correctly stated that antibiotics kill bacteria. Children from households where the caretaker answered the question “When your child’s throat hurts, do you give them antibiotics?” with “sometimes” had lower odds of ESBL-EC carriage than those with a caretaker response of “never” (OR 0.48, 95% CI 0.27–0.87).

**Conclusion:**

Caregivers in our study population generally demonstrated low knowledge regarding appropriate use of antibiotics. Our findings suggest that misinformation about the types of infections (i.e. bacterial or viral) antibiotics should be used for may be associated with elevated odds of carriage of ESBL-EC. Understanding that using antibiotics is appropriate to treat infections some of the time may reduce the odds of ESBL-EC carriage. Overall, however, KAP measures of appropriate use of antibiotics were not strongly associated with ESBL-EC carriage. Other individual- and community-level environmental factors may overshadow the effect of KAP on ESBL-EC colonization. Intervention studies are needed to assess the true effect of improving KAP on laboratory-confirmed carriage of antimicrobial resistant bacteria, and should consider community-level studies for more effective management.

## Background

The World Health Organization and the U.S. Centers for Disease Control and Prevention have identified extended-spectrum β-lactamase producing Enterobacteriaceae (ESBL-Ent) as a serious threat to public health [1]. The prevalence of these bacteria varies across the globe. A recent study in Argentina estimated the prevalence of ESBL-Ent carriage to be 19% of community members [2], and another study in Spain found carriage at 16% [3]. The global prevalence has been estimated to be approximately 14%, ranging from 2 to 46% among individuals, depending on the region [4].

South America has been identified as a region with high rates of ESBL-Ent, but data characterizing the magnitude of the problem remains lacking in Ecuador [5, 6]. Recently, ESBL-producing *Escherichia coli* (ESBL-EC), commonly used as an indicator for ESBL-Ent, was found in 6% of Gram-negative bloodstream infections tested in Quito [7], indicating that further research in Ecuador is warranted to understand the extent of the problem [8]. This study aimed to advance the knowledge of the drivers for ESBL-EC carriage in Ecuador, in addition to examining risk factors that may influence carriage worldwide.

An individual’s knowledge, attitudes, and practices (KAP) towards proper use of antibiotic agents may influence the risk of ESBL-EC colonization in that individual, and potentially among their household members or children. Understanding whether KAP can influence colonization is important, as pathways for antimicrobial resistance (AMR) and carriage of resistant bacteria are likely affected by knowledge of and attitudes towards antibiotic agents, which determine practices associated with antibiotic use.

KAP studies are typically applied in clinical settings to assess individual beliefs and behaviors, and how people engage in health-affecting behaviors. Many studies have used KAP of proper use of antibiotic agents as an outcome, analyzing demographic data as the risk factors. These studies have used a wide range of populations, such as farmers [9], community members [10, 11], and medical students [12]. While these studies identify avenues for intervention, they do not directly link KAP to the laboratory-confirmed presence or absence of drug-resistant bacteria. To the authors’ knowledge, only three studies to date have analyzed the link of KAP to laboratory-confirmed outcomes: (1) KAP and presence of antibiotic resistant, diarrheagenic *E. coli* [13]; (2) KAP of pig farmers and AMR carriage in their pigs [14], and (3) knowledge of proper antibiotic use in Singaporeans and AMR carriage among that population [15]. The present study builds on these previous studies and aims to assess how KAP of proper use of antibiotic agents is associated with laboratory-confirmed carriage of antibiotic resistant bacteria. Specifically, we set out to investigate whether a lack of KAP regarding proper use of antibiotic agents among the primary caretakers of children was a risk factor for their children being colonized by ESBL-EC.

## Methods

### Recruitment

The study was carried out among 444 households over two cycles of visits with six months of time between each visit. Households were chosen at random from six peri-urban parishes of Quito, Ecuador, provided they met the inclusion criteria. Inclusion criteria for the households were: (1) households with a primary child caretaker present who was over 18 years of age, (2) a child between the ages of 6 months and 5 years old present in the household; and (3) informed consent provided by the caretaker to participate in the study. Stool samples were obtained from 374 children at the first visit (July to November 2018). During the second visit (January to May 2019), we attempted to sample the same 374 children. If the family was lost to follow-up, we enrolled new households that met the same inclusion criteria. Two-hundred ninety-six households participated in both timepoints, 78 were lost between cycle one and cycle two, and 70 new households were added for cycle two to maintain statistical power.

### Sample collection

One fecal sample was collected from each enrolled child for each cycle and tested for presence of ESBL-EC. The children’s stool samples were collected by the child’s primary caretaker. Caretakers were given oral and written instructions on how to collect the fecal sample and deposit into the provided container. Participants were instructed to keep the sample container double-bagged in the refrigerator until field staff could pick the sample up the same morning. The samples were transported to the laboratory in a cooler kept on ice and were processed within eight hours of collection.

### Survey data collection

An Ecuadorian field team administered the survey to the caretakers at each home at the time of sample collection for each cycle. Sections of the survey included demographic characteristics, health, and environmental health information, as well as a section on caretakers’ KAP of proper use of antibiotic agents. Eleven questions regarding KAP were asked. Questions were derived from those posed in other KAP-based studies [11, 16] and were discussed and modified with local experts to ensure that the questions would be appropriate for the study’s specifications. Surveys were first developed in English, translated to Spanish, and then translated back to English. The interview team field-tested the surveys to determine culturally appropriate and relevant questions. The surveys were adjusted based on feedback during the first four weeks of sampling. Surveys were collected by the trained field team and results were digitized using Open Data Kit software. Results were then exported to R for data analysis.

### *E. coli* isolation

Upon arrival at the lab, fecal samples were streaked onto MacConkey agar (Difco, Sparks, Maryland) with the third-generation cephalosporin ceftriaxone (2 mg/L) and incubated at 37 °C for 18 h. Up to five lactose positive colonies phenotypically matching *E. coli* were preserved at − 80 °C in Trypticase Soy Broth with 20% glycerol for later antibiotic susceptibility testing. If less than five colonies grew from the fecal sample, all were selected, in order to obtain as many strains as possible.

### Antibiotic susceptibility testing

To test for antibiotic resistance, up to two isolates for each child at each cycle were thawed and regrown on MacConkey agar at 37 °C for 24 h for evaluation of antibiotic susceptibility by the disk diffusion method (Kirby Bauer test) on Mueller–Hinton agar (Difco, Sparks, Maryland). Frozen bacteria were first re-plated on MacConkey agar and incubated at 37 °C for 24 h. Once regrown, strains confirmed as *E. coli* were plated onto Mueller Hinton agar for antibiotic susceptibility testing [17]. To confirm the isolates were *E. coli,* colonies were inoculated onto Chromocult® Coliform agar (Merck, Darmstadt, Germany)*.* Chromocult® coliform agar has been shown to have high sensitivity and specificity for *E. coli* isolates [18]. Further testing on 5% of the Chromocult®-confirmed isolates was performed with the API 20E test (bioMérieux, France) for quality control. There were two isolates per child, unless only one isolate grew.

Isolates were identified as either susceptible or resistant to each antibiotic according to the resistance or susceptibility interpretation criteria from Clinical and Laboratory Standards Institute (CLSI) guidelines [19]. *E. coli* ATTC 25922 was used as the quality control strain. Antibiotics used for susceptibility testing included: amoxicillin-clavulanic acid (AMC; 20/10 μg), ampicillin (AMP; 10 μg), cefazolin (CFZ; 30 μg), cefotaxime (CTX; 30 μg), ceftazidime/clavulanic acid (CAZ/CLA; 30/10 μg), ceftazidime (CAZ; 30 ug), cefepime (FEP; 30 μg), ciprofloxacin (CIP; 5 μg), gentamicin (GEN; 30 μg), imipenem (IPM; 30 μg), tetracycline (TET; 30 µg) and trimethoprim-sulfamethoxazole (SXT; 1.25/23.75 μg) [19].

Results from the antibiotic susceptibility test were used to determine if the *E. coli* were ESBL-producing or multidrug-resistant. To determine if the *E. coli* were ESBL-producing, the combination disk diffusion test was used with CAZ and CAZ/CLA as outlined in the CLSI and EUCAST guidelines [20]. Isolates were considered multidrug-resistant if they were resistant to three or more macro-classes in addition to the antibiotic ceftriaxone, here defined as cephalosporin/b-lactamase inhibitors, penicillins, aminoglycosides, carbapenems, fluoroquinolones, tetracyclines, and folate pathway inhibitors [21].

### Statistical analysis

The ESBL-EC prevalence was calculated as the proportion of children with a fecal sample testing positive for an ESBL-EC-producing isolate out of all children with a fecal sample. Multidrug-resistant bacteria prevalence was calculated as the proportion of individuals with a fecal sample that produced an isolate resistant to three or more classes of antibiotics. Stool samples that showed no growth on the initial MacConkey agar plate with ceftriaxone were counted as non-ESBL-EC producing.

Binomial logistic regressions were used to determine Odds Ratios (ORs) for KAP survey results and carriage of ESBL-EC and multidrug-resistant *E. coli.* To adjust for repeated measures at each household, we used generalized estimating equations for robust variance estimation and an exchangeable working correlation due to unbalanced data. A univariate analysis was performed on each of the KAP survey variables. To avoid dropping individuals from the analysis who did not answer every KAP question, non-answers were grouped with the “do not know” response.

Following univariate analyses, multivariate analyses were performed with KAP variables and a priori confounding variables. Confounders of the association between KAP and ESBL-EC carriage were determined through literature review [4, 9, 11, 22–25]. A directed acyclic graph was used to determine which variables identified in the literature and found in the survey data would be included as confounders in the models. Confounder variables included in all the models were: the caregiver’s education level, household wealth, a family member working in or visiting a health care center in the six months before the survey, use of antibiotics among animals, and use of antibiotics by any individual in the household in the three months before the survey. All confounders were included in the multivariate regression, and distributions for each variable can be seen in Table 1. Regressions were performed for ESBL-EC carriage, multidrug-resistant *E. coli*, and fluroquinolone-resistant *E. coli*.

**Table 1:** Demographic characteristics of survey respondents by cycle

	Median or No. (%)	
	Cycle 1	Cycle 2
Median age of survey respondent (years) (n = 374, n = 366)	27	27
Sex of survey respondent (n = 374, n = 366)		
Female	344 (92.0)	333 (91.0)
Male	28 (7.5)	32 (8.7)
Other	2 (0.5)	1 (0.3)
Median size of households	4	4
Median number of children under 12 in household	2	2
Ethnicity (n = 374, n = 366)		
Mixed	329 (88.0)	342 (93.4)
Indigenous	18 (4.8)	11 (3.0)
Other	27 (7.2)	13 (3.6)
Highest education level of survey respondent (n = 371, n = 362)		
Primary education	91 (24.5)	93 (25.7)
Pre-secondary education	245 (66.0)	227 (62.7)
Secondary or higher	35 (9.4)	42 (11.6)
Wealth Index Score (n = 374, n = 366)		
Low	129 (34.5)	120 (32.8)
Medium	114 (30.5)	193 (52.7)
High	131 (35.0)	53 (14.5)
Animal ownership, including pets, livestock, or poultry (n = 371, n = 366)		
Yes	238 (64.2)	246 (67.2)
No	135 (36.4)	120 (32.8)
Administered antibiotics to animals in previous 6 months (n = 365, n = 362)		
Yes	42 (11.5)	37 (10.2)
No	323 (88.5)	325 (89.8)
Family member treated with antibiotics in past 3 months (n = 374, n = 366)		
Yes	42 (11.2)	45 (12.3)
No	332 (88.8)	321 (87.7)
Household member worked in or visited healthcare facility in 6 months prior to survey (n = 370, n = 365)		
Yes	44 (11.9)	26 (7.1)
No	326 (88.1)	339 (92.9)
Correctly answered both questions on whether antibiotics can kill viruses or bacteria (n = 372, n = 364)		
Yes	30 (8.1)	28 (7.7)
No	342 (91.9)	336 (92.3)
ESBL prevalence	44 (11.8)	41 (11.2)
MDR prevalence	161 (43.0)	151 (41.3)

Household wealth was determined by running a principal component analysis (PCA) with imputation for missing data on the household asset data to create a wealth index score [26], which was categorized by tertiles. The PCA included the following ownership variables to create the index: house, land, television, cable television, car, computer, and internet. Other confounding variables were used as coded in the survey data. Logistic regression models were used to determine adjusted ORs for the KAP variables. All statistical analyses were conducted in R (ver. 3.5.3). Generalized estimating equation (GEE) analysis was performed with the package *geepack* [27]*.*

## Results

### Demographic characteristics of the study population

We analyzed 740 stool samples from 444 children living in households. Among the households participating in the study, 296 participated in both cycles. Seventy-eight were lost from the first cycle and replaced by 70 new households for the second cycle. Three-hundred and seventy-four households responded to the survey and gave fecal samples at cycle one, and 366 households responded to the survey and gave fecal samples at cycle two. Demographic characteristics of the respondents can be seen in Table 1. Over both cycles, the survey respondents were a majority female, of mixed ethnicity, with at least a primary education. Distribution of the demographics generally remained the same across both cycles, with the exception of the wealth index tertiles, which is reflective of asset ownership and may change between cycles. Only six children were found to be ESBL-EC positive at both timepoints.

### Responses on knowledge, attitudes, and practices

Knowledge about antibiotics in this study population was low. Only 8% of survey respondents correctly identified antibiotics as killing bacteria but not viruses (see Table 2). When asked about the effect of antibiotics on bacteria and viruses separately, less than half of respondents correctly stated that antibiotics can kill bacteria (40.8%), and only 158 respondents correctly stated that antibiotics do not kill viruses (21.4%). Knowledge about antibiotics’ effectiveness for a cold was also low, with only 22% of respondents correctly answering that antibiotics would not help a child with a cold improve faster. Attitudes towards proper antibiotic use were mixed, with half of respondents saying it is sometimes ok to use antibiotics when sick to help one feel better (50.8%). Three hundred ninety-three respondents said they always wait or hope for the doctor to prescribe antibiotics when they are sick (53.1%).

**Table 2:** Frequency of KAP responses

Question	No. of responses (%)
When your child’s throat hurts, do you give them antibiotics? (n = 740)	
Never	179 (24.2)
Sometimes	280 (37.8)
Always	259 (35.0)
Don’t know/no response	22 (3.0)
When your child catches a cold, will antibiotics help them improve more quickly? (n = 740)	
Never	162 (21.9)
Sometimes	193 (26.1)
Always	333 (45.0)
Don’t know/no response	52 (7.0)
When your child is sick with a cold, bad enough that they need to see a doctor, do you wait for a doctor to prescribe antibiotics for your child? (n = 740)	
Never	124 (16.8)
Sometimes	206 (27.80
Always	393 (53.1)
Don’t know/no response	17 (2.3)
Do you think it is OK to use antibiotics when you feel sick, to help you get better? (n = 740)	
Never	115 (15.5)
Sometimes	376 (50.8)
Always	225 (30.4)
Don’t know/no response	24 (3.2)
If your child has bronchitis and you cannot take them to the doctor quickly, is it an option to go to a pharmacy to get antibiotics? (n = 740)	
Never	432 (58.4)
Sometimes	136 (18.4)
Always	148 (20.0)
Don’t know/no response	24 (3.2)
Do most of your friends think that they should give antibiotics to their children when they have a cold? (n = 740)	
Never	159 (21.5)
Sometimes	168 (22.7)
Always	200 (27.0)
Don’t know/no response	213 (28.8)
Do most of your friends think that they should give antibiotics to their children when they have diarrhea? (n = 740)	
Never	156 (21.1)
Sometimes	159 (21.5)
Always	199 (26.9)
Don’t know/no response	226 (30.5)
Do most of your friends think that they should give antibiotics to children when they have skin irritations? (n = 740)	
Never	156 (21.1)
Sometimes	163 (22.0)
Always	188 (25.4)
Don’t know/no response	233 (31.5)
Do most of your friends go to a pharmacy to buy antibiotics for their children who are ill, without a medical prescription? (n = 740)	
Never	142 (19.2)
Sometimes	156 (21.1)
Always	213 (28.8)
Don’t know/no response	229 (30.9)
Can antibiotics kill bacteria? (n = 740)	
Yes	302 (40.8)
No	124 (16.8)
Don’t know/no response	314 (42.4)
Can antibiotics kill viruses? (n = 740)	
Yes	280 (37.8)
No	158 (21.4)
Don’t know/no response	302 (40.8)
Correctly answered both questions on whether antibiotics can kill viruses or bacteria (n = 736)	
Yes	58 (7.9)
No	678 (92.1)

Of the KAP categories, practice had the highest amount of protective responses. Only 35.0% of respondents said they always give antibiotics to their child when their throat hurts. Most respondents were also unable to buy antibiotics from the pharmacy without first going to the doctor (58.4%). Descriptive norms—beliefs and behaviors people normally engage in which can affect behaviors of others—were also studied among this population, by asking respondents what their friends’ attitudes and practices towards antibiotic use were. As seen in Table 2, the answer distribution was relatively even among responses, with the highest response for each question being “don’t know/no response”. Investigation of a pattern found by exploratory multiple correspondence analysis revealed that the respondents generally answered all four descriptive norms questions with the same answers.

### Resistance patterns

The prevalence of ESBL-EC among confirmed *E. coli* isolates growing on MacConkey agar with ceftriaxone was 95 isolates out of 516 (18.4%) (see Table 3). There was a high prevalence of multidrug-resistant isolates as well—86.4% of the Ceftriaxone-resistant bacterial isolates were resistant to three or more classes of antibiotics. Carbapenem resistance was seen in three isolates (0.6%). Over 99% of the isolates were resistant to Cefazolin and Ampicillin.

**Table 3:** Phenotypic resistance of *E. coli* isolates by study cycle

Class	Antibiotic	Cycle 1 No. of isolates (%)^a^ n = 277	Cycle 2 No. of isolates (%) n = 239	Total No. of Isolates among both cycles (n = 516)
Cephalosporin/β-lactamase inhibitor	Cefazolin	277 (100%)	238 (99.6)	515 (99.8)
	Ceftazidime	72 (26.0)	58 (24.3)	130 (25.2)
	Cefepime	121 (43.7)	106 (44.4)	227 (44.0)
	Cefotaxime	256 (92.4)	227 (95.0)	483 (93.6)
	Amoxicillin/clavulanic acid	63 (22.7)	43 (18.0)	106 (20.4)
Penicillin	Ampicillin	275 (99.3)	238 (99.6)	513 (99.4)
Aminoglycoside	Gentamycin	47 (17.0)	47 (19.7)	94 (18.2)
Carbapenem	Imipenem	2 (0.7)	1 (0.4)	3 (0.6)
Fluroquinolone	Ciprofloxacin	137 (49.5)	118 (49.4)	255 (49.4)
Tetracycline	Tetracycline	201 (72.6)	187 (78.2)	388 (75.2)
Folate pathway inhibitors	Trimethoprim-Sulfamethoxazole	185 (66.8)	157 (65.7)	342 (66.3)
ESBL-producing		52 (18.8)	43 (18.0)	95 (18.4)
Multidrug-resistant to three or more classes^b^		231 (83.4)	217 (90.8)	448 (86.4)

Antibiotic susceptibility testing was performed on bacteria strains that had previously grown on MacConkey agar with ceftriaxone. Percentages represent percent of isolates tested

^a^Strains tested were determined to be the same isolate if they came from the same fecal sample and had the same resistance pattern

^b^Multidrug resistant *E. coli* were determined from isolates resistant to ceftriaxone

### KAP and ESBL-EC

The association between the KAP questions and carriage of ESBL-EC was determined by GEE analysis at both the univariate and multivariate level. Results showed a significant association for three KAP questions (Table 4). For the question “When your child’s throat hurts, do you give them antibiotics?” the answer “sometimes” had an adjusted OR of 0.48 (95% CI 0.27–0.87) when compared to the answer “never”. The question “Can antibiotics kill bacteria?” had an adjusted OR of 2.17 (95% CI 1.18–3.99) for the answer “no” compared to the answer “yes”. The question “Can antibiotics kill viruses?” had an adjusted OR of 0.56 (95% CI 0.32–0.97) for the answer “yes” and an adjusted OR of 0.52 (95% CI 0.30–0.90) for the answer “don’t know”, compared to the answer “no”. The remaining KAP questions did not have significant associations with the outcome of ESBL-EC carriage when adjusting for confounding. One of the confounders, education level, was found to be positively correlated with good KAP (data not shown). Regressions were also run looking at the association between KAP and multidrug-resistant *E. coli*, and the association between KAP and fluoroquinolone-resistant *E. coli*, but no meaningful associations were found (data not shown).

**Table 4:** KAP risk factor analysis for ESBL *E. coli* carriage in children

Question	ESBL positive (%)	ESBL negative (%)	OR univariate (95% CI)^b^	*P* value	OR adjusted (95% CI)^c^	*P* value
*When your child’s throat hurts, do you give them antibiotics?*						
Never	28 (15.6)	151 (84.4)	Ref		Ref	
Sometimes	23 (8.2)	**257 (91.8)**	**0.48 (0.27–0.86)**	**0.01**	**0.48 (0.27–0.87)**	**0.02**
Always	29 (11.2)	230 (88.8)	0.68 (0.38–1.20)	0.18	0.65 (0.36–1.15)	0.14
Don’t know/no response^a^	5 (22.7)	17(77.3)	1.45 (0.45–4.70)	0.53	1.64 (0.45–5.99)	0.45
*When your child catches a cold, will antibiotics help them improve more quickly?*						
Never	21 (13.0)	141 (87.0)	Ref		Ref	
Sometimes	23 (11.9)	170 (88.1)	0.90 (0.48–1.68)	0.74	0.82 (0.42–1.59)	0.55
Always	34 (10.2)	299 (89.8)	0.77 (0.42–1.39)	0.39	0.70 (0.38–1.30)	0.26
Don’t know/no response	7 (13.5)	45(86.5)	1.06 (0.40–2.80)	0.91	1.00 (0.36–2.75)	1.00
*When your child is sick with a cold, bad enough that they need to see a doctor, do you wait for a doctor to prescribe antibiotics for your child?*						
Always	41 (10.4)	352 (89.6)	Ref		Ref	
Sometimes	24 (11.7)	182 (88.3)	1.11 (0.65–1.90)	0.69	1.11 (0.64–1.93)	0.71
Never	17 (13.7)	107 (86.3)	1.35 (0.76–2.40)	0.31	1.38 (0.77–2.46)	0.28
Don’t know/no response	3 (17.6)	14 (82.3)	1.74 (0.36–8.4)	0.49	1.94 (0.35–10.71)	0.45
*Do you think it is OK to use antibiotics when you feel sick, to help you get better?*						
Never	17 (14.8)	98 (85.2)	Ref		Ref	
Sometimes	39 (10.4)	337 (89.6)	0.65 (0.36–1.19)	0.16	0.61 (0.33–1.11)	0.10
Always	26 (11.6)	199 (88.4)	0.73 (0.39–1.35)	0.31	0.66 (0.35–1.24)	0.19
Don’t know/no response	3 (12.5)	21 (87.5)	0.74 (0.15–3.76)	0.72	0.72 (0.13–3.99)	0.70
*If your child has bronchitis and you cannot take them to the doctor quickly, is it an option to go to a pharmacy to get antibiotics?*						
Never	52 (12.0)	380 (88.0)	Ref		Ref	
Sometimes	15 (11.0)	121 (89.0)	0.89 (0.49–1.62)	0.71	0.91 (0.49–1.67)	0.76
Always	134 (90.5)	14 (9.5)	0.76 (0.41–1.40)	0.38	0.78 (0.41–1.46)	0.43
Don’t know/no response	4 (16.7)	20 (83.3)	1.46 (0.42–5.12)	0.55	1.76 (0.47–6.59)	0.40
*Do most of your friends think that they should give antibiotics to their children when they have a cold?*						
Never	21 (13.2)	138 (86.8)	Ref		Ref	
Sometimes	20 (11.9)	148 (88.1)	0.85 (0.44–1.63)	0.63	0.84 (0.42–1.67)	0.61
Always	179 (89.5)	21 (10.5)	0.77 (0.42–1.40)	0.39	0.78 (0.42–1.45)	0.43
Don’t know/no response	23 (10.8)	190 (89.2)	0.79 (0.43–1.45)	0.46	0.77 (0.41–1.43)	0.40
*Do most of your friends think that they should give antibiotics to their children when they have diarrhea?*						
Never	20 (12.8)	136 (87.2)	Ref		Ref	
Sometimes	21 (13.2)	138 (86.8)	1.02 (0.54–1.92)	0.96	1.07 (0.55–2.07)	0.85
Always	22 (11.1)	177 (88.9)	0.85 (0.45–1.58)	0.60	0.89 (0.47–1.69)	0.72
Don’t know/no response	22 (9.7)	204 (90.3)	0.74 (0.40–1.38)	0.35	0.75 (0.40–1.41)	0.37
*Do most of your friends think that they should give antibiotics to children when they have skin irritations?*						
Never	21 (13.5)	135 (86.5)	Ref		Ref	
Sometimes	20 (12.3)	143 (87.7)	0.87 (0.46–1.64)	0.66	0.91 (0.46–1.79)	0.78
Always	21 (11.2)	167 (88.8)	0.81 (0.44–1.51)	0.51	0.84 (0.44–1.59)	0.59
Don’t know/no response	23 (9.9)	210 (90.1)	0.71 (0.38–1.30)	0.27	0.71 (0.38–1.33)	0.29
*Do most of your friends go to a pharmacy to buy antibiotics for their children who are ill, without a medical prescription?*						
Never	20 (14.1)	122 (85.9)	Ref		Ref	
Sometimes	16 (10.3)	140 (89.7)	0.67 (0.33 –1.36)	0.27	0.70 (0.34–1.48)	0.36
Always	29 (13.6)	184 (86.4)	0.98 (0.53–1.78)	0.94	1.04 (0.56–1.96)	0.89
Don’t know/no response	20 (8.7)	209 (91.3)	0.59 (0.31–1.12)	0.11	0.60 (0.31–1.16)	0.13
*Can antibiotics kill bacteria?*						
Yes	29 (9.6)	273 (90.4)	Ref		Ref	
No	23 (18.5)	**101 (81.5)**	**2.18 (1.20–3.97)**	**0.01**	**2.17 (1.18–3.99)**	**0.01**
Don’t know/no response	33 (10.5)	281 (89.5)	1.12 (0.67–1.90)	0.66	1.09 (0.64–1.85)	0.75
*Can antibiotics kill viruses?*						
No	26 (16.5)	132 (83.5)	Ref		Ref	
Yes	29 (10.4)	251 (89.6)	0.59 (0.34–1.04)	0.07	**0.56 (0.32–0.97)**	**0.04**
Don’t know/no response	30 (9.9)	272 (90.1)	**0.56 (0.32–0.98)**	**0.04**	**0.52 (0.30–0.90)**	**0.02**

Significant associations are bolded

^a^Non-responses to the survey were decided to be included with those not knowing their response and were included in the model in order to avoid dropping individuals missing data for single questions, for a total of 740 responses

^b^The answer deemed as the knowledge, attitude, or practice that is best or is hypothesized to most limit antibiotic use serves as the referent category

^c^The questions included in the model as confounders can be found in the methods section

## Discussion

### Association between KAP and ESBL-EC carriage

This study found low KAP of appropriate antibiotic use among the study population. Most participants had low knowledge on the function of antibiotics, and this was in some cases significantly associated with carriage of ESBL-EC*.* Only 8% of respondents were aware that antibiotics are effective for bacteria, but not effective for viruses, indicating low awareness in the population about the function and use-cases for antibiotics. This demonstrates a potential need for educational campaigns to better inform the population of the reasons for using different types of medications.

Children from households where the caregiver incorrectly stated that antibiotics do not kill bacteria had higher odds of ESBL-EC carriage than those in households where caregivers correctly stated that antibiotics kill bacteria (OR 2.17, 95% CI 1.18–3.99). This suggests understanding the relationship between antibiotics and bacteria could be an important piece of knowledge to target when aiming to reduce AMR. Knowledge of the effects of antibiotics on viruses also had a significant association with ESBL-EC carriage. Children whose caregivers incorrectly stated that antibiotics kill viruses or stated that they did not know the answer to this question had lower odds of ESBL-EC carriage than those who answered correctly (OR 0.56 and 0.52, respectively). This goes against the hypothesis that correct knowledge of antibiotic function is protective against AMR. The association found could be due to respondents being unaware of the difference between bacteria and viruses. Further research in this population is necessary to better understand the driving factors of these associations. In addition, knowledge does not always translate to practice. When answers from both questions were combined, no association was seen between knowledge of antibiotic function and ESBL-EC carriage. This may be due to the small number of respondents (8%) answering both questions correctly.

The knowledge levels among this study population are comparable to those found by Mo et al. in Singapore. Their study found that 43.8% of their population believed antibiotics were for viral infections [15], while 37.8% of this study population believed antibiotics can kill viruses. However, the effect of knowledge on ESBL-EC carriage differed between both studies. Mo et. al found a higher knowledge score associated with higher odds of ESBL-EC carriage [15], while our study found associations between ESBL-EC carriage and knowledge to be mixed. Our study also saw a similar level of knowledge on the effect of antibiotics on viruses as a multi-country public awareness survey performed by the World Health Organization. In their survey of twelve countries, 64% of respondents affirmatively stated that antibiotics can treat cold or flu, instead of answering with “don’t know” or “no” [28], while 71.1% of respondents in our survey said that antibiotics will always or sometimes help their child improve from a cold more quickly.

We assessed descriptive norms, the beliefs and behaviors that one believes their friends and neighbors normally engage in [29] as they apply to proper antibiotic use, and found little variability between each question. Descriptive norms have been found to influence behavior in a wide range of areas, from littering to alcohol use [30, 31]. Using norms as an intervention point in AMR has been shown to have an effect on prescribing rates [32], encouraging further investigation in this field. In our study, respondents generally answered the four norms questions of the survey with the same response. This may indicate that the respondents only have a general sense of others’ KAP and are not informed to the granular level posed by the individual questions. When treating all four questions as one variable, no association was found between the descriptive norms and ESBL-EC carriage.

Only one antibiotic practice variable was associated with ESBL-EC carriage in children. “Sometimes using antibiotics for a sore throat” was significantly associated with lower odds of carriage than never using antibiotics to treat a sore throat (OR 95% CI 0.48, 0.27–0.87, see Table 4). “Sometimes using antibiotics” was also associated with lower odds when compared to always (data not shown). While never using antibiotics was hypothesized to be the response that would most reduce the odds of ESBL-EC carriage, sometimes using antibiotics may be a better representation of proper antibiotic use. It could be that those who answered “sometimes” may rely on a doctor’s opinion more than others and recognize there are some indications for antibiotics, such as streptococcal angina, and therefore may have better antibiotic use practices.

Beyond the question about the practice of using antibiotics for a sore throat and the questions regarding knowledge of antibiotic function, other KAP questions did not see an association with carriage. This is similar to the results found by GebreSilasie et. al. in the context of child diarrhea. They found that of the questions assessing knowledge, attitudes, and practices of diarrhea, only knowledge scores had an association with diarrheagenic *E. coli* carriage, while practice and attitude scores did not have an association [13].

Overall, most questions relating to KAP of proper antibiotic use did not find associations with carriage of ESBL-EC*.* While other studies have found an association between non-rational use of antibiotics and increased AMR carriage [14], this study did not. The lack of association may reflect a lack of awareness of the respondents’ own KAP of antibiotic use, since many answered “don’t know” to the questions. Associations may also be affected by residual confounding. It could also be that an association between risk factors and ESBL-EC carriage does not manifest at the individual level and is overshadowed by environmental factors.

Studies have shown that environmental factors more likely to have a community-wide effect appear to be associated with carriage of ESBL-producing and multidrug-resistant bacteria. In a study performed in Peru, Kalter et al. found that living in a population where a larger proportion of households ate home-raised instead of market-bought chicken was protective against AMR [24]. Improper waste management from farms and sanitation processes have been linked to AMR in the environment [33], which could impact carriage of AMR among this study population. Farming practices have also been found to influence carriage of AMR [34]; small-scale farms are common in the study population area. It could be that in this population, these larger-scale, environmental factors may outweigh the effect of KAP at the individual level.

Further investigations could examine KAP of proper antibiotic use and antibiotic-resistant bacteria carriage between different communities or neighborhoods, and determine whether KAP at a community and environmental level affects carriage of drug-resistant bacteria. Beyond community-level risk factors, studies have shown that acts such as international travel can affect carriage of AMR [35, 36], indicating that carriage of multidrug-resistant bacteria is not necessarily dependent on antibiotic use. This may also explain why more associations were not seen. Indeed, other studies have found that multidrug-resistant bacteria carriage is not necessarily associated with antibiotic exposure [37].

### ESBL-EC prevalence

The 11% prevalence of ESBL-EC aligns with the range seen in the literature. Studies of ESBL-EC prevalence in healthy volunteers have found it to range from 7–20% [38, 39]. Prevalence of multidrug-resistant *E. coli* among this study population (41.3–43.0%) also aligned with the range seen in the literature (21.3–49.0%), although the definition of MDR varies between two and three antibiotic classes [24, 40].

In addition to ESBL-EC and ceftriaxone-resistant multidrug-resistant *E. coli* prevalence being estimated among the total study population, they were also determined at the isolate level, along with other resistance patterns. The almost-ubiquitous resistance to Cefazolin and Ampicillin among the isolates that grew for 42% of the children indicates potential for treatment failure if doctors in this population prescribe ampicillin or cefazolin for illnesses. The identification of three isolates resistant to Imipenem is concerning, as carbapenems are considered last-line drugs.

One limitation of this study is that it may underestimate the level of multidrug resistance among the children in this region. In order to isolate any ESBL-EC, if present, and avoid picking up only dominant bacterial strains instead of ESBL-EC—which may or may not be dominant in the gut—the stool samples were plated on plates containing 2 mg/L ceftriaxone. While this made it possible to obtain the most complete picture of ESBL-EC carriage, it did bias the isolates that grew towards having multidrug-resistance, as they were already resistant to ceftriaxone. Multidrug-resistant isolates susceptible to ceftriaxone may have been missed. Even with this underestimate, the high level of multidrug resistance shows that measures to curb the spread of antimicrobial resistance in this population are warranted.

Future directions of research include determining if the state of KAP in this population changes over time. Beyond this study, more research into assessing the relationship of KAP of antibiotic use and the outcome of antibiotic-resistant bacteria carriage is necessary. Only three other studies to date have made this connection, each focusing on different aspects. From this small pool of data, it is difficult to determine if KAP can influence bacterial carriage, especially multidrug-resistant bacteria. Regardless of the association, the results of this study show that knowledge is low in this population, and that an educational campaign promoting better knowledge of antibiotics and prudent use may be a worthwhile endeavor.

This is an important area of study as improving KAP is a feasible pathway for intervention in low-income communities and has been done with success for different diseases [41, 42]. KAP interventions specifically focused on AMR in clinical settings and the community have also been shown to be effective [43–45]. While most of these interventions target providers, this study provides evidence that interventions should be tested at the community level as well.

## Conclusion

The rapid increase and global spread of ESBL-EC is an important public health issue. The present study assessed the relationship of child caretakers’ KAP towards proper antibiotic use with ESBL-EC carriage among children in a peri-urban community of Quito, Ecuador. Correct knowledge of antibiotics and their proper use was lacking among the study population, while the prevalence of ESBL-EC was similar to that found in other studies. Overall, there was not a strong association between KAP and ESBL-EC carriage, but significant associations were seen for ESBL-EC carriage at the knowledge level. Believing that antibiotics do not kill bacteria was associated with higher odds of ESBL-EC carriage, and a protective association was also seen between proper treatment of a sore throat and ESBL-EC carriage. The results indicate that there may be a connection between KAP of proper antibiotic use and ESBL-EC carriage, and that more studies linking laboratory-confirmed carriage and knowledge, attitudes, and practices of antibiotics are warranted. Studies assessing KAP and ESBL-EC carriage across multiple communities, as well as environmental determinants of antimicrobial resistance transmission, would help to further understand risk factors for ESBL-EC carriage.

## Data Availability

The data and code that support the findings of this study will be available from the corresponding author upon reasonable request.

## Abbreviations

ESBL-Ent: Extended-spectrum β-lactamase producing Enterobacteriaceae
ESBL-EC: ESBL-producing *E. coli*
KAP: Knowledge, attitudes, and practices
AMR: Antimicrobial resistance
AMC: Amoxicillin-clavulanic acid
AMP: Ampicillin
CFZ: Cefazolin
CTX: Cefotaxime
CAZ/CLA: Ceftazidime/clavulanic acid
CAZ: Ceftazidime
FEP: Cefepime
CIP: Ciprofloxacin
GEN: Gentamicin
IPM: Imipenem
TET: Tetracycline
SXT: Trimethoprim-sulfamethoxazole
OR: Odds ratio
GEE: General estimating equation
